# Development partner influence on domestic health financing contributions in Senegal: a mixed-methods case study

**DOI:** 10.1093/heapol/czae110

**Published:** 2024-11-21

**Authors:** Frederik Federspiel, Josephine Borghi, Elhadji Mamadou Mbaye, Henning Tarp Jensen, Melisa Martinez Alvarez

**Affiliations:** Department of Global Health and Development, London School of Hygiene and Tropical Medicine, 15-17 Tavistock Place, London WC1H 9SH, United Kingdom; Department of Global Health and Development, London School of Hygiene and Tropical Medicine, 15-17 Tavistock Place, London WC1H 9SH, United Kingdom; International Institute for Applied Systems Analysis, Schlossplatz 1, Laxenburg A-2361, Austria; Department of Political Science, Gaston Berger University, Route de Ngallèle, Saint Louis BP 234, Senegal; Institut de Recherche en Santé, de Surveillance Epidemiologique et de Formation (IRESSEF), Arrondissement 4 Rue 2 D1, Pole Urbain de Diamniadio, Dakar BP 7325, Senegal; Department of Global Health and Development, London School of Hygiene and Tropical Medicine, 15-17 Tavistock Place, London WC1H 9SH, United Kingdom; Department of Food and Resource Economics, University of Copenhagen, Rolighedsvej 23, Frederiksberg 1958, Denmark; Department of Global Health and Development, London School of Hygiene and Tropical Medicine, 15-17 Tavistock Place, London WC1H 9SH, United Kingdom; Independent consultant

**Keywords:** external development partners, donors, development assistance, equity, health financing, Senegal, interviews

## Abstract

Sustainable and equitably contributed domestic health financing is essential for improving health and making progress towards Universal Health Coverage (UHC) in low- and middle-income countries. In this study, we explore the pathways through which development partners influence the combination of domestic health financing sources in Senegal. We performed a qualitative case study that comprised 32 key stakeholder interviews and a purposive document review, supplemented by descriptive statistical analysis of World Health Organization and Organization for Economic Cooperation and Development data on health financing sources in Senegal. We developed a novel framework to analyse the different mechanisms and directions of development partner influence on domestic health financing contributions. We identified development partner influence via four mechanisms: setting aims and standards, lobbying/negotiation, providing policy/technical advice, and providing external financing. Overall, development partners worked to increase tax-based government contributions and expand Community-Based Health Insurance (CBHI), which is seemingly equity enhancing. Fungibility and intrinsic equity issues related to CBHI may, however, limit equity gains. We encourage stakeholders in the health financing sphere to use our framework and analysis to unpack how development partners affect domestic health financing in other settings. This could help identify dynamics that do not optimally enhance equity and support progress towards UHC to help achieve more coherent policy-making across all domains of development partner activities in support of UHC. Future research should investigate the role of international creditors, lending, and loan conditionalities on domestic health financing in recipient countries, including equity implications.

Key messages

- External development partners (EDPs) exert influence over domestic health financing policy in many low- and middle-income countries; however, their mechanisms of influence are poorly understood.
- Using a mixed-methods case study of Senegal, we found that EDPs use aim- and standard-setting, lobbying/negotiation, policy/technical advice, and external financing to influence domestic health financing sources.
- We found EDPs in Senegal used these mechanisms of influence to promote increases in domestic government health spending (Government Health Expenditure as a Source (GHE-S)) and Community-Based Health Insurance (CBHI) while seeking to reduce out-of-pocket payments (OOPs).
- However, while CBHI expanded, GHE-S saw limited real-term increases, and OOP prevailed in Senegal between 2000 and 2021.
- Our analytical framework can be used to explore EDP influence on domestic health financing sources in other contexts to identify areas of policy incoherence and support unified policy-making in pursuit of universal health coverage.

## Introduction

Equity in health financing contributions has long been recognized as essential to improving health indicators and making progress towards Universal Health Coverage (UHC) in low- and middle-income countries (LMICs), protecting patients and their families from financial risk (Makinen et al. 2000, Marmot 2007, Xu et al. 2007, WHO 2024b). We define equity of health financing contributions as funds being contributed in proportion to ability to pay; being prepaid, so funds can be made available to those who need it irrespective of their ability to pay at the time of seeking a health service; and being pooled across many individuals to allow for financial risk sharing (Wagstaff et al. 1989, Kutzin 2000, McIntyre and Mooney 2007, Mills et al. 2012, Mtei et al. 2012, Kutzin et al. 2017, Mathauer et al. 2019, Martinez-Alvarez et al. 2020, WHO 2024b).

Many recipient countries of development assistance are highly donor-dependent for their health sector financing (WHO 2024b), and development partners exert a great influence over national health policy (Parkhurst et al. 2018, Sparkes et al. 2019, Ridde and Faye 2022), including health financing policy (Kajula et al. 2004, Sridhar and Gomez 2011, Colenbrander et al. 2015, Gautier and Ridde 2017, Sparkes et al. 2019, Witter et al. 2019, Nagemi and Mwesigwa 2021, Alawode et al. 2022, Ridde et al. 2022, 2024a) in various contexts including Senegal. In a recent commentary by Ridde et al. (2024a), the authors explain how Community-Based Health Insurance (CBHI) in Senegal was pushed by external development partners (EDPs), stifling progress towards UHC by more than a decade due to poor coordination and intrinsic equity issues with CBHI.

In the quantitative literature, some econometric studies have examined the relationship between external and domestic financing in the form of fungibility, i.e. whether the health budget increases by less than the amount injected as development assistance for the health sector due to an associated decrease in Government Health Expenditure as a Source (GHE-S), i.e. from domestic revenue (Lu et al. 2010, Dieleman et al. 2013, Liang and Mirelman 2014, Patenaude 2021), with most authors finding a fungibility effect. Other studies have examined the relationship between external health financing and out-of-pocket payments (OOPs), finding no effect or a crowding-in effect (Xu et al. 2011, Younsi et al. 2016, Ali et al. 2020). However, these studies do not go beyond relationships between financing flows to explore the different potential pathways of development partner influence.

Understanding through which mechanisms development partners influence domestic health financing in aid recipient countries is important: this can help to inform development partner efforts, ensuring that they do indeed work towards sustainable, equitably contributed health financing in the countries they support.

Building on the aforementioned work and using the case of Senegal, our study aims to explore through which pathways development partners may influence the composition of domestic funding sources for health and whether this influence has been equity enhancing in the case of Senegal. We use qualitative methods (interviews and document review), supplemented by descriptive statistics to examine the presence, pathways, and nature of EDP influence on domestic health financing contributions, since 2000. We first describe the main financing sources and mechanisms in Senegal. We then introduce and apply a novel analytical framework to identify and examine the different ways development partners may exert influence on domestic health financing contributions in Senegal.

## Materials and methods

### Equity definition

As explained earlier, we define equity of health financing contributions as funds contributed in proportion to ability to pay, which are prepaid and pooled. This implies cross-subsidies of health funds from rich to poor and from the healthy to the sick (McIntyre 2008, Goudge et al. 2012). With this definition, OOPs are considered least equitable, as they do not take into account a person’s ability to pay, they are not prepaid, and there is no pooling of funds (WHO 2024b). Health insurance is more equitable than OOP, but to a varying degree depending on the level of contributions made relative to the ability to pay of insurance pool members, the level of cross-subsidy from rich to poor, and the size of pools.

Government health financing is considered most equitable, as taxes are overall progressive, although Value Added Tax and some excise taxes can be regressive. An individual benefiting from a fully tax-funded health service experiences no personal financial cost at the point of care, and funding pools can be large enough to cover an entire nation’s population, resulting in maximal risk sharing and cross-subsidy across the income and wealth spectrums of a nation. We thus consider donor influence on the composition of funding sources towards more government financing and less OOP and support for pooled financing mechanisms over no pooling as equity enhancing.

### Analytical framework

**Figure 1.**
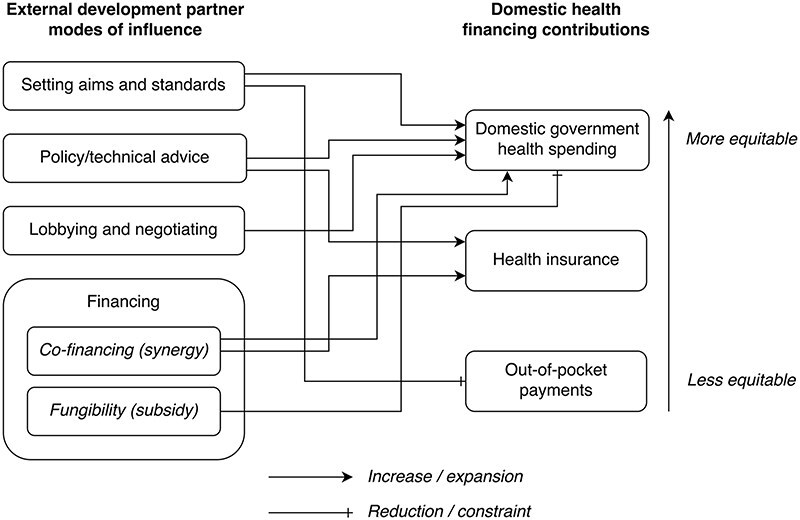
Framework for analysing EDP influence on domestic health financing contributions. Development partners can influence domestic health financing contributions via setting aims and standards, advising, lobbying and negotiating, and providing financing. Development partner financing may have a synergistic effect or a displacing effect on government health financing. These different mechanisms of influence may result in changes in the balance between different health financing sources—government health financing from taxes, health insurance premiums, and OOPs—which, in turn, has implications for the degree of equity of domestic health financing contributions. This figure is an original visualization based on the previous work of Steinberg (2003), Start and Hovland (2004), and De Raeve et al. (2022) and on the interview, document, and quantitative findings of our study in Senegal. The arrows reflect our findings in Senegal.

The framework used in this study for analysing development partner influence on domestic health financing contributions is illustrated in Fig. 1. We developed the framework iteratively, both inductively and deductively. We first conducted a literature review to identify existing policy influence analysis frameworks from the broader policy analysis literature, including health policy analysis frameworks. Existing frameworks have focused on, e.g. the nature of problems, politics, and policy (Kingdon 1984); context, policy content, policy process, and actors (Walt and Gilson 1994); policy-making processes (Grindle and Thomas 1991, Gautier and Ridde 2017); agents of policy change (Lindquist 2001); or the ideas, institutions, and interests underlying policy influence and reform (Hall 1997, National Collaborating Centre for Healthy Public Policy 2014, Mulvale et al. 2017). Others have emphasized political context, existing evidence, and links/network factors, (Crewe and Young 2002, Start and Hovland 2004, Overseas Development Institute (ODI) 2014); mechanisms of policy influence more broadly (Start and Hovland 2004, Cathexis Consulting 2015, De Raeve et al. 2022); and the role of international science and finance in determining LMIC policy (Steinberg 2003). A framework developed by Sparkes *et al*. for analysing the political economy of health financing reform has focused on the different dimensions of politics, e.g. bureaucratic politics, leadership politics, or external politics (Sparkes et al. 2019). Fox and Reich (2015) have combined Hall’s 3-i framework (Hall 1997) and Kingdon’s stream model (Kingdon 1984) to analyse how politics affects UHC reform in LMICs at different stages of the policy cycle (Fox and Reich 2015, Rizvi et al. 2020).

Three of the identified frameworks were found to emphasize policy influence mechanisms (Steinberg 2003, Start and Hovland 2004, De Raeve et al. 2022). These were adapted and adjusted into one framework that reflected the dimensions of EDP influence present in our data. We then applied the framework to our data, making any final adjustments needed based on the findings.

We found that development partners can exert influence on domestic health financing contributions via setting aims and standards, providing policy/technical advice, lobbying and negotiating, and providing finance. Development partner financing may elicit or require co-financing by the recipient government or displace government funds (fungibility/subsidy). These different modes of influence may result in changes in the balance between different health financing sources, which, in turn, affect the degree of equity of domestic health financing contributions (Kutzin 2000, McIntyre and Mooney 2007, Kutzin et al. 2017, WHO 2024b).

The influence mechanisms via giving policy advice or technical advice, and by lobbying and negotiating, are derived from Start and Hovland (2004) and De Raeve et al. (2022). Policy and technical advice can be viewed as evidence-based knowledge production and dissemination, often enacted through publishing official reports and briefings, allowing for the ‘diffusion of sector-specific know-how’ offering solutions to policy problems (Start and Hovland 2004, Galanti 2020, De Raeve et al. 2022). Lobbying/negotiating can be thought of as the art of persuasion, often involving high-level networking through people-to-people interactions in both formal and informal settings (Start and Hovland 2004, UK Parliament 2024).

The mechanism of providing external health financing is derived from Steinberg (2003). Based on our study results, we have added the policy influence mechanism: ‘setting aims and standards’, meaning influence through the establishment of aspirational concepts such as the Sustainable Development Goals or (evidence-based) best practice recommendations. We have further separated external financing into that which has an increasing or a displacing effect on domestic government health financing.

### Study setting

Senegal is a Francophone democratic republic in West Africa with a population of 18 million in 2023 [Agence nationale de la statistique et de la demographie (ANSD) 2023]. Classified as a lower-middle-income country, its Gross Domestic Product (GDP) per capita was $1599 in 2022 (World Bank 2024).

#### Overview of health financing sources in Senegal

**Figure 2.**
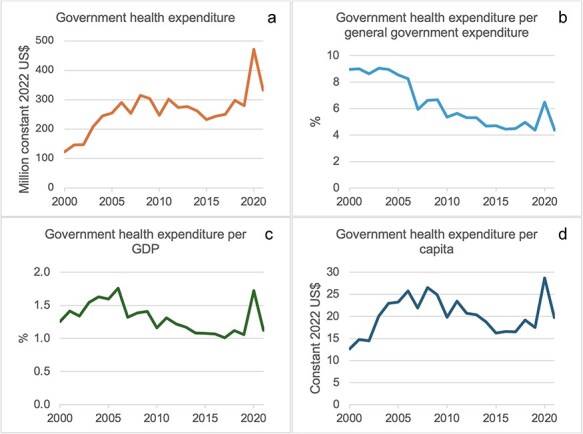
Government health expenditure from domestic revenue in absolute terms (a), as a proportion of GGE (b), as a proportion of GDP (c), and per capita (d) (World Bank 2024, WHO 2024b). (Constant US$ data deflated using WB US$ GDP deflator data (World Bank 2024)).

**Figure 3.**
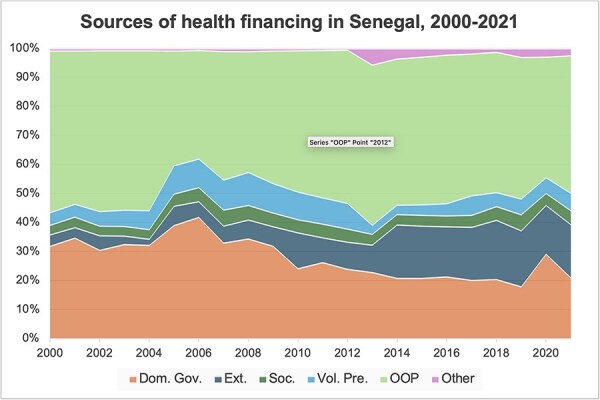
Sources of health financing in Senegal as percent of current health expenditure, 2000–21 (WHO 2024b). Dom. Gov: domestic government revenue; Ext: external financing; Soc: social insurance contributions; Vol. Pre: voluntary prepayment. Visualization method as per WHO (2024b). Notes: There may be some additional government health financing captured within Soc. and Vol. Pre. as subsidies to these schemes.

Over the past two decades, domestic health financing in Senegal has been characterized by a strong reliance on user contributions and a smaller and decreasing reliance on government contributions, with the exception of the year 2020 when government health financing saw a transient increase due to the Coronavirus disease 2019 (COVID-19) outbreak (Figs 2 and 3). Following a period of steady rise from 2000 to 2006, GHE-S was $290 million in 2006 and $280 million in 2019 (constant 2022 US$) (Fig. 2) (World Bank 2024, WHO 2024b). This corresponds to a per capita decrease from $26 in 2006 to $18 in 2019 (constant 2022 US$) (World Bank 2024, WHO 2024b) and a decrease per GDP from 1.8% in 2006 to 1.1% in 2019 (WHO 2024b) (Fig. 2). Before the COVID-19 pandemic, GHE-S also received decreasing budget priority, declining as a share of General Government Expenditure (GGE) from 8% in 2006 to 4% in 2019, getting further from the Abuja target of 15% (WHO 2024b) (Fig. 2). Following a period of relative decline from 2004 to 2006, OOP contributions correspondingly made up a growing proportion of all health financing in Senegal, from their lowest point in 2006 at 37% to reach 49% in 2019 (Fig. 3) (WHO 2024b). This corresponds to a per capita increase from $21 in 2006 to $36 in 2019 (constant 2022 US$) (World Bank 2024, WHO 2024b).

Partly mitigating the previous lack of growth in domestic government health financing, real-term external health financing has expanded substantially from $14 million in 2000 to $236 million in 2021 (constant 2022 US$) (World Bank 2024, WHO 2024b). This corresponds to an increase from 4% to 18% of current health expenditure over the same time period (Fig. 3) or from $1 to $14 per capita (constant 2022 US$) (World Bank 2024, WHO 2024b).

**Figure 4.**
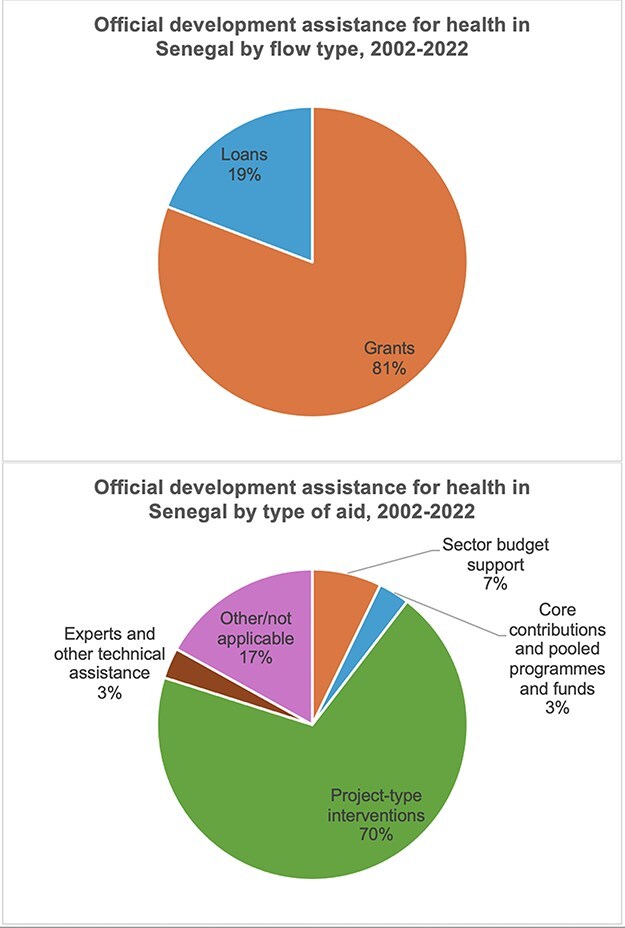
Total ODA for health in Senegal disbursed between 2002 and 2022, separated by flow types and types of aid (OECD 2024).

As seen in Fig. 4, total official development assistance (ODA) for health disbursed between 2002 and 2022 has been provided predominantly as grants (81%) and as project-type interventions (70%) (OECD 2024).

#### Health financing schemes

Various health financing schemes exist in Senegal, as summarized in Table 1. The current composition of health financing schemes in Senegal is strongly influenced by the 2014 ‘Plan Sénégal Émergent’ (‘Emerging Senegal’) that charted a course for all sectors, including health sectoral reform (Government of Senegal 2014). Currently, it mainly consists of a set of exemption schemes for vulnerable groups, priority services, and drugs called the *Gratuités*; compulsory health insurance schemes for formal sector workers and their families; and CBHI schemes called the *Mutuelles* for all Senegalese though mainly targeting informal sector workers and the rural poor (Agence de la Couverture Maladie Universelle 2024, Daff et al. 2020, Division des Institutions de Prevoyance Maladie 2021, ICAMO 2023, Ministère de la Santé et de l’Action Sociale (MSAS) 2017b, Ministère du développement communautaire, de l’equité sociale et territoriale 2021, Paul et al. 2020, Wood 2023).

**Table 1.** Overview of health financing schemes in Senegal (Agence de la Couverture Maladie Universelle 2024, Daff et al. 2020, Division des Institutions de Prevoyance Maladie 2021, ICAMO 2023, Ministère de la Santé et de l’Action Sociale (MSAS) (2017b), Ministère du développement communautaire, de l’equité sociale et territoriale 2021, Paul et al. 2020, Wood 2023)

Scheme type	Scheme name	Target groups	Funding source	Pooling
Exemptions	*Gratuités*	People >60 years, children <5 years, caesarean sections, dialysis, antiretroviral and tuberculosis drugs	Government contributions, donor contributions	National level
Compulsory health insurance	*Assurance Maladie Obligatoire (Imputation budgetaire, Institutions de Prévoyance Maladie, and more)*	Civil servants + families, formal sector employees + families, retired state and private sector employees + families, university students, occupational injury and illness coverage and more	Member contributions, employer/organization contributions, private donations	Scheme members
CBHI	*Mutuelles de santé*	All Senegalese, though mainly informal sector workers, rural poor	Member contributions, state contributions,^a^ private donations	Members, community level^a^
Private health insurance	Various	Anyone, though mainly wealthier groups	Member contributions	Scheme members

a For most of those enrolled in *Mutuelles*, the state pays 50% of the nationally fixed annual premium of 7000 Communauté Financière Africaine francs per person (Daff et al. 2020, Wood 2023) (∼US$12), while certain very poor or disabled groups can obtain 100% subsidy (Ministère de la Santé et de l’Action Sociale (MSAS) 2017b; Paul et al. 2020; Wood (2023)). *Mutuelles* are currently undergoing consolidation from the community to the departmental level (Daff et al. 2020, Ridde et al. 2022, 2024a).

### Study design, sampling, data collection, and management

This study was a qualitative case study comprising key stakeholder interviews and a purposive document review, supplemented by descriptive quantitative analysis of health financing in Senegal. Interviews were conducted in Senegal between October 2019 and January 2020. Documents and quantitative data were collected before, during, and after this period and analysed after an initial analysis of interviews.

### Purposive document review

**Figure 5.**
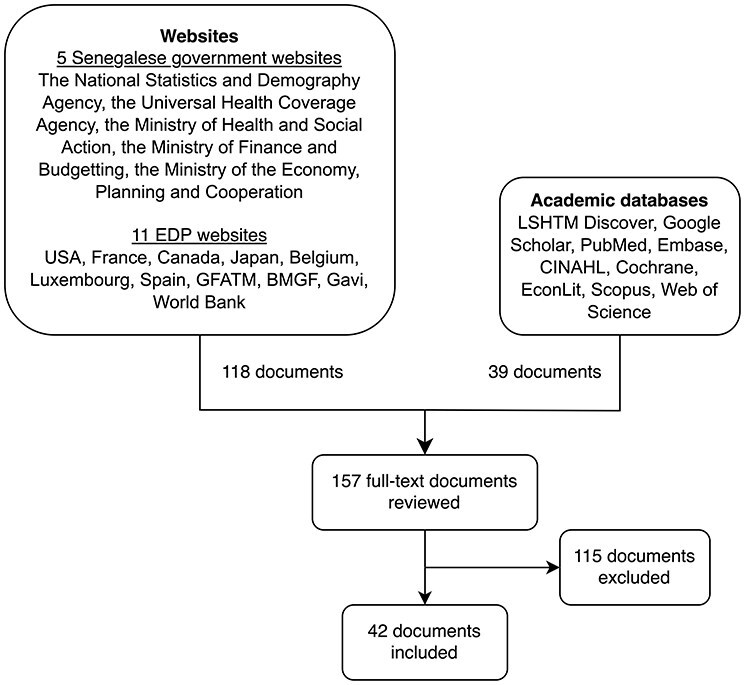
Flowchart for purposive document review. LSHTM: London School of Tropical Medicine & Hygiene.

**Figure 6.**
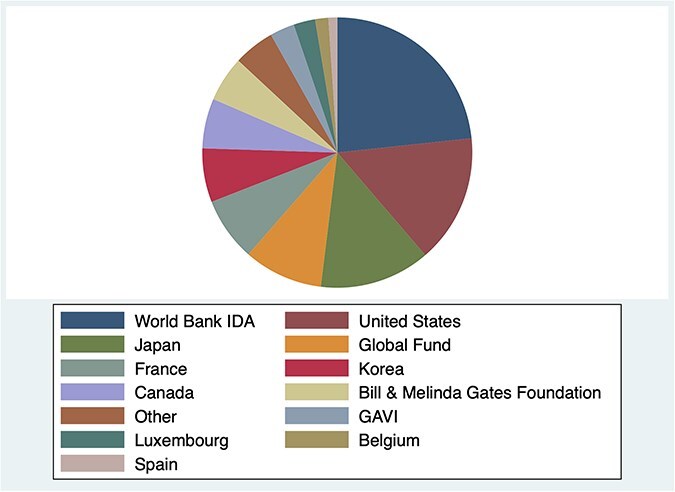
Main official development partners in the health sector in Senegal by disbursements made in 2022 (OECD 2024). ‘Other’ sums disbursements from 28 multilateral organizations, bilateral organizations, and private foundations, each less than $3 million.

To further investigate EDP influence on domestic health financing sources, we searched government and development partner websites for articles and official reports on health financing and health financing policy in Senegal. This was done by screening websites from 11 EDPs and the Senegalese government for available links and references that could potentially discuss health financing (e.g. ‘our work’ => ‘global health’) (Fig. 5). Websites from the following organizations were screened: the Senegalese government (the National Statistics and Demography Agency; the Universal Health Coverage Agency; the Ministry of Health and Social Action; the Ministry of Finance and Budgeting; and the Ministry of the Economy, Planning and Cooperation), the major bilateral and multilateral donors present in Senegal (USA, France, Canada, Japan, Belgium, Luxembourg, Spain, the Global Fund to Fight Aids, Tuberculosis and Malaria (GFATM), Bill and Melinda Gates Foundation (BMGF), and Gavi) (OECD 2024) (Fig. 6), as well as the World Bank (WB). We also searched Google Scholar, PubMed, Embase, Cochrane, Cumulative Index to Nursing and Allied Health Literature (CINAHL), Scopus, Web of Science, and EconLit for relevant academic literature, using keywords including Senegal, health financing, and equity. A total of 157 full-text articles and reports were retrieved for full-text review (118 from organization websites and 39 via academic databases). Articles/reports documenting development partner activities with direct implications for domestic health financing contributions or providing facts confirming/rebutting cited statements from interviews were included and integrated into the Results section. Forty-two out of the 157 full-text documents reviewed were included in the results. No time period constraints were applied to the document review in order to also obtain a broader historical understanding of EDP activities and health financing policy in Senegal; however, we focus our results on the period after 2000.

### Semistructured interviews

We conducted an initial mapping of key external and domestic stakeholders engaged in health financing in Senegal by searching websites and through discussion with contacts in academia and government. Representatives from the main international official donors, government ministries and agencies, Senegalese civil society organization leaders and academics, and management and administrative staff at the regional, district, and hospital levels were included. Once the initial set of stakeholder institutions and persons were identified, snowball sampling was used to identify additional participants (Table 2). We also included participants from the region of Tambacounda, as this region is the largest geographical region in the country with an estimated population of nearly 1 million people in 2023 (Agence nationale de la statistique et de la demographie (ANSD) 2024) and has one of the highest poverty rates in the country at 62% in 2018/19 (Agence Nationale de la Statistique et de la Démographie 2021). Repetition of similar observations and positions became apparent towards the end of interview data collection, indicating that data saturation was reached (Saunders et al. 2018).

**Table 2.** Interview participants

	Number of participants		
Stakeholder group	Total	Dakar	Tambacounda
Bilateral development partners	4	4	0
Multilateral development partners	4	4	0
Ministries/central government agencies^a^	9	9	0
Regional/district health management	3	0	3
Hospital management	4	1	3
Civil society organizations	5	4	1
Academics	3	3	0
Total interviews	32	25	7

a Participants from ministries/central government agencies came from four different ministries/agencies; however, the specific ministry/agency is intentionally not mentioned to protect the anonymity of participants.

We conducted 32 interviews, 25 at the national level in Dakar and 7 at the district and regional level in Tambacounda (Table 2). Representatives from two multilaterals, two hospitals, and two academics were unavailable/did not respond. We used an interview topic guide to elicit the participant’s organization’s activities, views/positions, and observations relevant to the composition and degree of equity of health financing in Senegal and development partner influence hereon. Informed consent was obtained from all participants. Interviews were recorded where consent for this was given (*n = *27). In the five instances where interviews were not recorded, F.F. took written notes and the interviews were used to broadly further his understanding of the research topic in Senegal and of the interviewees’ general views on this (see the ‘Limitations’ section). Interviews lasted from about 30 minutes to just over an hour. Thirty interviews were conducted face-to-face, and two were conducted remotely. Interviews were conducted in the preferred language of the participant. In most instances, this was French, and in some instances, this was English. Interview materials were provided in the corresponding language. A professional interpreter was used for the majority of French-language interviews until F.F. had reached adequate proficiency for conducting interviews in French independently. Interviews were transcribed by a professional transcriptionist. Both the interpreter and the transcriptionist were fluent French/English bilingual Senegalese professionals, both with bachelor’s degrees from the USA. F.F. controlled the quality and validity of transcripts by comparing segments from all interviews with the transcripts, including all instances of inaudibility/lack of clarity. Interviews were analysed and interpreted in their original language. Written notes were taken from all interviews. All interviews were treated as anonymous. The participant information sheets, informed consent forms, and an example interview topic guide can be found in the Supplementary Appendix.

#### Interview data analysis

Interviews were analysed using the framework method (Ritchie and Spencer 1994). We used NVivo for interview coding (QSR International Pty Ltd 2020). We developed our coding framework based on a combination of deduction of predetermined themes and induction of themes from the data. F.F. first coded a third of all interviews in an exploratory manner to establish themes (*n = *11), ensure conceptual clarity, and avoid overlap or omission of themes present within the data (Ritchie and Lewis 2003). F.F., J.B., and M.M.A. then agreed on the final coding framework. An independent researcher then co-coded a transcript for validation, after which the final coding framework was applied to all transcripts. Summaries and central/illustrative quotations were entered into the framework matrix. The final dataset was then systematically reviewed for patterns and relevant opinions, factual statements, and explanatory accounts.

### Quantitative data

We used the WHO Global Health Expenditure Database (GHED) (WHO 2024a) to perform descriptive quantitative analysis of health financing sources (GHE-S, External Health Financing (EXT), OOP, and Voluntary Health Insurance (*Mutuelles*)). We looked at trends in the composition of health financing sources over time from 2000 to 2020 (all available data) to contextualize and triangulate information from our other sources.

## Results

We identified partner influence on domestic health financing contributions via four mechanisms: setting aims and standards, lobbying/negotiation, providing policy/technical advice, and providing external financing (Fig. 1). Our findings generally indicated an equity-promoting role of development partners in regard to domestic contributions; however, concerns were raised as to their actual effect as government health funding cuts had been observed.

### Setting aims and standards

Commenting on the slow growth in government health spending seen in Senegal, seven out of eight development partner representatives interviewed stated that they wanted to see stronger increases in the government health budget, with several referring to the Abuja target of domestic government health expenditure, making up at least 15% of GGE (Organisation of African Unity 2001). This desire was echoed across all stakeholder groups, including ministerial/government agency representatives.

The country signs agreements, and in these agreements, it is asked to make a budget that is approved for health that must reach 15%. This is an external pressure, and the country is bound to make efforts to achieve this. (Ministry/government agency)

The Abuja Declaration comes from African nations themselves (Organisation of African Unity 2001), but donors used this as a normative standard towards which they wanted the Senegalese government to aspire. An internally derived aim thus became a partly externally promoted aim. However, over the period 2006–19, there was no discernible increase in real-term government health financing, while GHE-S/GGE decreased from about 8% to 4% (Fig. 2). This indicates that this normative/aspirational influence pathway from both EDPs and African nations jointly has been ineffective in Senegal during this time period.

An academic also referred to the 1978 Alma Ata Declaration (WHO 1978) as another international standard used to promote UHC.

We have now the universal health coverage, is it coming from Senegal? No Senegal has to implement it because we signed it. In 1978 when the world decided on primary health care, we signed it and we started implementing. (Academic)

Overall, there was mostly universal agreement among interviewee stakeholder groups, including donors, that Senegal should aim to reduce OOP. In terms of their overall policy stance, most donor representatives interviewed stated that they wanted the future development of Senegal’s health sector to be characterized by higher government contributions and less reliance on OOP:

[Donor] encourages the countries to work on means to reduce user fees at service delivery points. Those are barriers in accessing health services and we very much support implementation of measures that facilitate access to health services by all populations, especially the poor population. (Donor)

These positions follow the 2017 Senegalese National Health Financing Strategy (Ministère de la Santé et de l’Action Sociale (MSAS) 2017a) and the 2019 National Plan for Health and Social Development (Ministère de la Santé et de l’Action Sociale (MSAS) 2019) and are as such consistent with official government policy.

As will be elaborated further, an academic also highlighted the WB publication ‘Investing in Health’ as influential on the Senegalese government in promoting primary health care financing, which indicates external aim-/standard- or norm-setting by the WB (World Bank 1993).

Development partners and government interviewees uniformly viewed Senegal as a nation with a high level of sovereignty and self-governance, setting its own targets with development partners supporting those targets:

In an organized country (Senegal), where there is a benchmark that serves as a reference, a partner cannot come and invest just anywhere… We are the ones who send funding requests to partners… The funding that is requested is always within the framework of what we want in terms of priorities. (Ministry/government agency)

Some interviewees in academia and civil society, however, disagreed with this view, arguing that donor funding priorities dictated government health programme priorities:

Each partner comes with their priorities, and the state in order to have the financing accepts everyone’s priorities… Usually the priority is dictated by the funder. (CSO)

For this study, we did not identify any partner documents externally setting binding standards or aims for domestic health financing in Senegal, consistent with views expressed by government and donor representatives (Ministère de la Santé et de l’Action Sociale (MSAS) 2017a; Ministère de la Santé et de l’Action Sociale (MSAS) 2019).

### Lobbying/negotiation and policy/technical advice

Statements of lobbying, negotiating, or ‘pushing’ as well as providing policy/technical advice for increased government health financing were given by some development partner representatives and academics. Policy/technical advice supporting UHC and CBHI was described in partner documents as well.

To help the Ministry of Health (MoH) attain a higher budget and support its execution, a donor gave both technical and negotiation support as follows:

What we are supporting is the planning process of the budget formulation. So, we are supporting the minister of health in the negotiation with the ministry of economy and finance…for additional resources in the health sector… We are also trying to support the execution of the budget… Training of some officials in the ministry of health about the procedures and the requirements of the budget execution. (Donor)

This suggests external support in internal negotiations to mobilize more government funds for health, thus encompassing dimensions of both technical advice and negotiation. Referring to UHC, an academic described technical advice received from the WHO:

Senegal cannot really isolate itself and say no I’m not listening to the world experts… You decide on the basis of advice that the international donors are advising. The technical guidance should be all of us, should be behind WHO whose mandate is to orient, guide and support our countries. (Academic)

They also described the WB ‘pushing’ the Senegalese government to view health spending as an investment:

In 2004 [original publication 1993], the World Bank published a document, that inspired our government which is Investing in Health… For the first time, the World Bank found that investing in health has a return… It helped… When they said investing in health, they started pushing the government to invest more money in primary healthcare which was good. (Academic)

This relates both to aim-/standard-setting as described earlier, but this was then described as followed by a ‘push’ (categorized as lobbying/negotiation) by the WB towards the Senegalese government once this new aim had been established.

Partner websites and reports listed several examples of efforts to expand domestic health financing contributions by strengthening CBHI, as described further. We categorize these as policy/technical advice. The United States Agency for International Development (USAID) and the United Nations International Children’s Emergency Fund (UNICEF) were helping the Senegalese government develop and implement their national health financing strategies to expand UHC and *Mutuelles* (UNICEF 2020; USAID 2023). WB support for the *Couverture Maladie Universelle* and *Mutuelles* included technical advice, e.g. ‘supporting new institutional arrangement to promote greater efficiency in internal processes of the UHI [“Couverture Maladie Universelle” or UHC] scheme’ (World Bank 2019, 2020). The Global Financing Facility (GFF) provided ‘technical support on developing a Theory of Change to further inform implementation of the Investment Case…’, which includes consolidation of *Mutuelle* risk pools (Global Financing Facility 2022).

### Financing

Providing health financing was identified as a key way development partners sought to influence domestic health financing contributions. These findings generally illustrate development partners seeking to increase government health financing and expand and consolidate CBHI. This can be seen as equity enhancing by better aligning payments with the ability to pay in the case of increased government health financing and to some degree for *Mutuelles* given that 50%–100% of premiums are paid for by the state. It also shows attempts to consolidate health insurance pooling at the departmental level, which increases financial risk sharing and cross-subsidization, although with disagreement between partners along the way (Jean Hugues 2018, Ridde et al. 2024a).

USAID, the French Development Agency [Agence Française de Développement (AFD)], and the WB provided external support for the rollout of *Mutuelles* across the country during the past decade (Alenda-Demoutiez 2017; Fonteneau et al. 2017, Ministère de la Santé et de la Prevention 2009, Ministère de la santé et de la prevention 2010, World Bank 2019). Using a combination of loans and grants, the WB together with multiple donors gave financial support to the Senegalese government for strengthening the *Couverture Maladie Universelle* programme including the *Mutuelles* (World Bank 2019, 2020). This illustrates external financial support for strengthening domestic health financing schemes. Since 2014, there has, however, apparently been initial disagreement between partners about the need for consolidation of *Mutuelles*, with Enabel (Belgium) for and USAID and WB against (Deville 2018, Jean Hugues 2018, Ridde et al. 2024a). Informed by USAID-, WB- and Enabel-supported pilots of funding pool consolidation, the Senegalese government has begun moving financial risk pooling from the community level to the departmental level (Daff et al. 2020, Ridde et al. 2022, 2024a). External financing has thereby indirectly led to a consolidation of funding pools, which is equity enhancing. In 2019, the GFF partnership, consisting of France (AFD), Gavi, GFATM, GFF, Japan (Japan International Cooperation Agency), WB, USAID, UNICEF, and other United Nations agencies, provided a $140 million loan and $10 million grant to support ‘…the government’s commitment to increase the share of its health budget from 4% (of total government expenditure) to 10% by 2022’ [(Global Financing Facility 2022) (this number was 4% in 2019 and 6% in 2020 (WHO 2024b) (Fig. 2))], extending *Mutuelle* insurance premium exemption for the poorest members and aggregating *Mutuelle* pools at the departmental level. This shows external financial support for expansion of government health financing and CBHI.

#### Co-financing versus fungibility

Despite the aforementioned investments, some interviewees did, however, call into question whether financial support from development partners stimulated an increase in government health spending (co-financing) or a decrease (subsidy/fungibility) (Fig. 1).

##### Co-financing

We found that examples of donors leveraging government finance include GFATM, Gavi, and the United Nations Population Fund, which have government co-financing requirements for their health programmes (Jha et al. 2022, The Global Fund 2022). A donor explained:

[Donor] provides resources and the government has to provide also the cost share… The conditionalities are that you have to put at minimum 25% of the total envelope [Donor] is providing you (Donor).

Another example was the provision of $154 million from the WB to help co-finance the Senegalese government’s COVID-19 response in 2021 (World Bank 2023), during which a great increase in domestic government health financing was seen (Fig. 2).

##### Fungibility

Several of our interviewees claimed that development partner financing, however, led to decreases in government health spending, i.e. fungibility. Some government officials denied the presence of fungibility, while others believed that it took place.

Most of the time, when donors intervene, we are asked to give counterparts [i.e. co-financing], and we try to satisfy these counterparts. Without taking into account that we have to readjust… Especially when it comes to budget support, fungibility exists when it comes to budget support. (Ministry/government agency).

A donor representative described cuts to the health budget during the government fiscal year as an explanation for why government health spending has not increased significantly in Senegal, and the interviewee attributed these cuts to the high presence of donors in the health sector:

… What they [the government] did during the development of the budget at the beginning of the fiscal year, so they give the amount… At the middle of the year, they introduced what they call the amending finance law… And they cut the budget… In the health sector… Because there are more donors in the health sector. So that means that the donor resource funding influences the decision of the government in reducing the budget.. If they cut the budget, the first target population who will be impacted is the poor and vulnerable population. (Donor)

Of the 15 years since 2000 when amending finance laws were available, the government expenditure budget for the MoH was cut eight times compared to six times for the Ministry of Education (MoE) (Direction Générale du Budget 2024) (This citation covers 23 budget documents available via the link provided in the reference). On average, the MoH lost 0.3% of its initial budgets through these amendments, while the MoE gained 0.6% (Direction Générale du Budget 2024). Some expenditure for health and education, however, exists outside of these ministries. Furthermore, internal versus external revenue source for a given ministry’s spending is not delineated in these documents. These numbers also do not elucidate the drivers behind budget cuts, and whether the presence of donors plays a role as claimed is thus not possible to verify using our other data sources.

Another government official emphasized the positive effect of within-sector fungibility of development partner financing for health by freeing up government resources for other social/health purposes:

… Where partners put in a lot of resources, for example when we speak of certain priority diseases, we see that the state puts less resources… They indirectly influence domestic financing by permitting the state to put many more resources into neglected aspects (mentions social protection and NCDs) (Ministry/government agency).

**Figure 7.**
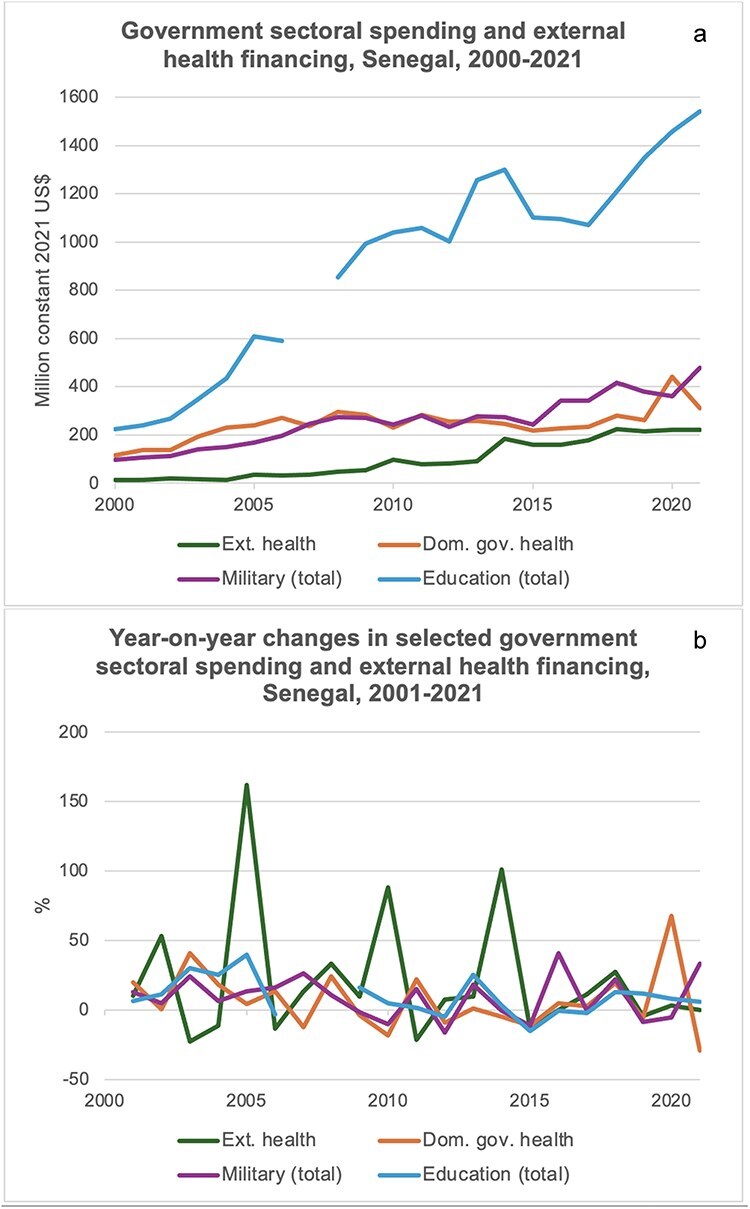
(a) Government spending for sectors with available data and external health financing in Senegal, 2000–21 (World Bank 2024, WHO 2024b; US$ GDP deflator used to transform current to constant US$ for all four categories, which leads to slightly lower health financing values than deflated WHO GHED values). (b) Real-term year-on-year changes in government spending for sectors with available data and external health financing in Senegal, 2001–21 (based on constant 2021 US$; World Bank 2024, WHO 2024b).

While these key-informant statements did not provide hard evidence for the presence of fungibility, which can be difficult to assert, they suggested fungibility as a potential mechanism constraining domestic government health financing. Fig. 7 displays real-term absolute levels and year-on-year changes in government spending for sectors with available data and external health financing between 2000 and 2021 (World Bank 2024, WHO 2024b). As illustrated, these time series do not allow for any judgement regarding the presence or absence of fungibility in the health sector, underlining the importance of key-informant observations.

## Discussion

The main objective of this study was to examine the pathways through which development partners influence the combination of domestic funding sources for health in Senegal. Our analysis identified four potential pathways of influence: setting aims and standards, lobbying/negotiation, providing policy/technical advice, and financing (Fig. 2). The influence identified generally appeared to be equity enhancing, mainly in terms of expanding government health financing, supporting existing insurance mechanisms, and promoting an increase in the size of insurance scheme risk pools. Development assistance fungibility was, however, identified by some key informants as a dynamic potentially dampening the level of domestic government health financing. Some of the identified pathways of development partner influence were similar to those reported in studies exploring broader EDP influence on recipient governments, including on health and health financing policy. Within gender equality promotion and migration control in Senegal, Olivie (2022) found consultants and people-to-people exchanges similar to our lobbying/negotiation and policy/technical advice, which tend to occur through such interactions. Olivie (2022) found infrequent presence of tied aid and no evidence of aid conditionality and attributed this to alignment between donor and government objectives. This resonates well with our finding that Senegal was generally seen to set its own development objectives and that partners aligned with these. Technical expertise and financing/financial incentives are frequently cited EDP modes of influence on health policy in other contexts, e.g. in Tanzania (Fischer and Strandberg-Larsen 2016), Uganda (Nabyonga Orem et al. 2013, Razavi et al. 2019), Pakistan, and Cambodia (Khan et al. 2018), and for health financing reform, e.g. in Ghana (Koduah et al. 2015), Nigeria (Onoka et al. 2015, Alawode et al. 2022), Pakistan (Khalid et al. 2024), and Thailand (Herberholz and Hotchkiss 2020). Lobbying has been conceptualized as a general mode of influence in the political economy of UHC reform in LMICs (Fox and Reich 2015), which our findings support in the case of EDPs in Senegal.

Some authors have emphasized normative power and the diffusion/transfer of international norms (Shiffman 2014, Bazbauers 2017), described by some as rooted in neoliberal ideals in the 1990s and enacted by the international financial institutions, and how this promoted marketization of health systems and limited government health spending in partner countries (Palier and Mandin 2009). This dimension was reflected in our aims and standards category. In Senegal, other authors have found that the use of CBHI as the primary instrument in the path towards UHC was influenced by a coalition of national and international actors, in part shaped by the ideas, institutions, and interests of EDPs (Deville 2018, Deville et al. 2018). On the allocation side of health financing, EDPs have been found to act as ‘diffusion entrepreneurs’, inducing diffusion of performance-based financing policy across Sub-Saharan Africa (SSA) (Gautier et al. 2018, 2019, Gautier 2019). As further confirmed by other authors (Kajula et al. 2004, Colenbrander et al. 2015, Gautier and Ridde 2017, Witter et al. 2019, Nagemi and Mwesigwa 2021, Alawode et al. 2022, Ridde et al. 2022, 2024a), the role of EDPs in shaping health financing policy in SSA across both contributions and allocations appears to be well supported, with our study shedding further light on influence mechanisms for health financing contributions. Some authors have also used the case of the international response against Human Immunodeficiency Virus, tuberculosis, and malaria in the 1990s and 2000s as examples of homogenous, vertical approaches in a heterogeneous Africa, with associated marginalization of African states (Eboko 2015, Nagemi and Mwesigwa 2021). Eboko (2015) saw hope for a return to agency, which we saw manifested across our interviews in Senegal, and signs of successful government ownership have been found in health financing policy reform across SSA, including for user fees exemption policies (Gautier and Ridde 2017). Future research could extend our findings and investigate the differential responsiveness to and integration of the different pathways identified on the part of recipient governments.

Our results did not provide examples of EDPs using public advocacy as a means of influence (Start and Hovland 2004, De Raeve et al. 2022). This is consistent with EDP influence occurring more in direct exchange with the Senegalese government rather than by advocating publicly. This may reflect a functional and intricate collaboration between the Senegalese government and its external partners, where appealing to the government indirectly is unnecessary for EDPs.

For the health sector, our findings illustrate how important it is for development partners to consider to what extent all their technical, political, and financial activities support partner governments in progressing towards improved equity of domestic health financing contributions and achieving UHC. There may be inconsistencies, where one branch of activities supports the government in mobilizing more funds for health, while another helps expand user-fee contributions or regressive insurance premiums. The identified health financing policy analysis frameworks do not specifically emphasize mechanisms or pathways of EDP policy influence, while the identified broader policy influence analysis frameworks focusing on mechanisms/pathways stem from the broader development space without specific application to health financing policy reform (Kingdon 1984, Grindle and Thomas 1991, Hall 1997, Lindquist 2001, Crewe and Young 2002, National Collaborating Centre for Healthy Public Policy 2014, Overseas Development Institute (ODI) 2014, Cathexis Consulting 2015, Fox and Reich 2015, Gautier and Ridde 2017, Mulvale et al. 2017, De Raeve et al. 2022, Rizvi et al. 2020, Sparkes et al. 2019, Start and Hovland 2004, Steinberg 2003, Walt and Gilson 1994). Our analytical framework fills this gap in the literature by focusing on the different pathways or mechanisms of EDP influence on different health financing sources. In doing so, the derived framework may help understand how the different main EDP activities pursue certain directions in the mix of domestic health financing sources. This may facilitate identification of areas of EDP policy incoherence on the path towards UHC. Once identified, this could form the basis for constructive discussion between government and EDP on how to address or minimize these. Due to a relatively high degree of sovereignty, political vision, and quality of policy formulation, we generally saw a high degree of donor alignment and thus analysed EDP influence jointly. This may, however, vary greatly across contexts. If applying our framework individually across multiple EDPs in a country (e.g. first the WB, then the WHO, etc.), this would allow the analyst to map out health financing policy incoherence, separated by EDP and by mechanism. This could potentially add a degree of nuance that could further the utility of our framework as a diagnostic tool for EDP health financing policy incoherence, helping to identify which branches of activities in which organizations promote reliance on which health financing sources. Next steps for further developing our analytical framework could be to integrate co-determinants arising from the domestic political economy, which would require new dedicated empirical enquiry. It is also conceivable that some pathways might be present in some countries but not in others, necessitating corresponding amendments. The financing pathway could also be further exploded into loans and grants and investigate the downstream effects of debt repayments arising from loans. Another avenue would be to integrate the ‘3-i’ framework (Hall 1997) analogous to that in the study by Fox and Reich (2015), exploring the underlying determinants for the EDP influence seen (see the ‘Limitations’ section).

In Senegal, development partners have provided their support of CBHI in the form of *Mutuelles*; however, the *Mutuelles* have been critiqued. Issues have included relying on user co-payment, creating relatively small pools with variable financial sustainability, limiting cross-subsidy from rich to poor and financial risk protection of poor members, and instituting voluntary enrolment with limited reach (Mladovsky and Ndiaye 2015, Daff et al. 2020, Ly et al. 2022, Ridde et al. 2023, 2024a, Wood 2023). Efforts to consolidate *Mutuelles* at the departmental level are ongoing (Ridde et al. 2023, 2024a), which, however, does not raise risk pooling to the national level (Jean Hugues 2018, Daff et al. 2020). Arguments for the decentralized CBHI model included management being rooted in communities with a higher degree of community ownership and the historical presence of CBHI in Senegal, leading to higher social acceptability (Deville 2018, Deville et al. 2018). Arguments for a joint departmental model have included administrative professionalization, improved risk pooling, efficiency, and financial viability (Deville 2018, Ridde et al. 2024a, 2024b). The proposed administrative centralization in the departmental model, enabling increased cross-subsidy among many more members, can be viewed as adhering more to a social welfarist ideology as opposed to the decentralized model, described by some as rooted in neoliberal ideology (Jean Hugues 2018). This exemplifies how differences in priorities and ideologies between partners can cause conflict in the search for a preferred UHC strategy. The value of EDP-supported pilots of alternative health financing mechanisms before broader scale-up, including CBHI, has been not only noted in Cambodia (Ir et al. 2010), in Ethiopia (Mulat et al. 2022), and across Low-Income Countries (Kiendrébéogo and Meessen 2019) but also critiqued as incoherent and ineffective in supporting health financing reform due to poor donor coordination and harmonization in Tajikistan (Jacobs 2019). Viewed together, this highlights the importance of the principles of effective development cooperation from the Paris Declaration, Accra Agenda, and Busan Partnership for successful EDP-supported health financing reform (OECD 2005, 2008, 2011, Kiendrébéogo and Meessen 2019).

Several interviewees identified development assistance fungibility as a mechanism limiting government health spending. The fungibility dynamic in development assistance for health is a well-described phenomenon (Farag et al. 2009, Lu et al. 2010, Stuckler et al. 2011, Xu et al. 2011, Dieleman et al. 2013, Fernandes Antunes et al. 2013, Dieleman and Hanlon 2014, Younsi et al. 2016), and while undesirable for donors, it has been viewed as rational redistribution of funds by others (Martinez Alvarez et al. 2016, Rana and Koch 2019). Most of the ODA for health since 2002 has been disbursed as project-type interventions (70%), which are more tightly ear-marked (OECD 2023), and only 7% has been disbursed as sectoral budget support, which limits the scope for fungibility of the injected funds themselves. Crowding-out of government funds is, however, still possible, if the government deems that externally funded projects cover certain population health needs and then decides, for whatever reason, to withdraw or not to supplement with funding for the same population health needs.

With the mentioned caveats of a possible fungibility effect and the small financial risk pools of decentralized CBHI, which is now being reformed, EDPs in Senegal generally appear to have been a force for improved equity of domestic health financing sources. They have used their identified influence pathways to promote a mix of domestic health financing sources characterized more by progressive, tax-based contributions from the government and less by OOP. In the complex political economy of UHC reform, the incrementalist approach, building on existing CBHI structures, may, however, have limited the overall scope for equity improvements compared to a more universalist approach (Fox and Reich 2015, Deville 2018, Ridde et al. 2024a). The enduring predominance of OOP in Senegal and limited real-term growth in GHE-S indicate that despite their efforts, EDPs have not been successful in achieving a more equitable domestic health financing mix. Differing interests from both different domestic stakeholders, including domestic policy makers and the Senegalese mutualist movement, as well as between different EDPs (USAID, Enabel and the WB), may have co-determined the limited progress seen over time (Deville 2018, Jean Hugues 2018, Ridde et al. 2024a).

### Limitations

The dynamics we have investigated in this study result from policy processes that often occur behind closed doors and are subject to unspoken ideology, power dynamics, and political considerations (Bachrach and Baratz 1962, Lukes 2005, Erasmus and Gilson 2008, Shiffman 2014, Anderson 2018). Interview participants may also have held incorrect or imprecise information, and causal pathways from EDP actions to domestic health financing impacts may be complex. Also, there is no counterfactual, and it is not possible to truly know how domestic health financing would have differed in the absence of development partner influence. These circumstances inevitably limited the extent to which we could access the ‘truth’ of our research question. We sought to mitigate this limitation by interviewing a broad range of stakeholders, offering them anonymity so they could speak freely, and using a range of other sources of information to triangulate statements. Future studies could search for natural policy experiments, possibly at the regional or district level, where comparable geographical entities are subjected to different EDP-supported health financing reforms, such as the Enabel-funded pilot of departmental aggregation of CBHI in Senegal. Such studies should, however, bear in mind the historical and present influence from other EDPs, and finding a true ‘untouched’ control seems improbable.

We were also unable to measure actual equity of financing through financing incidence analysis. Instead, we sought to provide an indication of whether and how development partners influenced financing mechanisms that are typically more or less equitable.

Our results did not allow us to explore the underlying reasons for ‘why’ development partners used a particular mechanism or tried to push health financing contributions in a certain direction. The ‘3-i’ framework by Hall (1997) (ideas, interests, and institutions) is one possible basis for approaching this question (Hall 1997, National Collaborating Centre for Healthy Public Policy 2014, Mulvale et al. 2017), as exemplified by Fox and Reich (2015), Deville (2018), Parkhurst et al. (2021), and Mhazo and Maponga (2022). On the allocation side, neoliberal ideology has been pointed out as a reason for development partners promoting performance-based financing in Senegal (Jean Hugues 2018). Future research should further interrogate the role of International Financial Institution policy recommendations and loan conditionalities in determining domestic health financing contributions and allocations in Senegal.

Certain nuances may have been lost in translation during interviews. To mitigate this, a professional interpreter was used for French-language interviews until this was no longer necessary. Interviews were also transcribed in their original language by a professional transcriptionist, so all nuances in wordings were retained in the data and could be interpreted *post hoc*. F.F. was, however, not fully proficient in French, which may have resulted in minor limitations in his ability to understand and interpret some linguistic nuances and subtleties. The interpreter was not involved in transcription of interviews or analysis of interview data to mitigate this. Where interviews were conducted in English as per the stated preference of the participant, this may still not have been their primary working language, and some depth and nuance may have been lost as a result. We acknowledge these limitations, which tend to be present in cross-language qualitative research (Squires 2009). The proficiency of our team in both French and English and the use of a professional interpreter and transcriptionist should, however, have rendered impacts on our results and interpretation from French to English translation altogether minimal.

Five participants did not allow the interview to be recorded or quoted directly in the paper, which greatly limited the analytical utility of these interviews. In an attempt to mitigate this effect, F.F. took notes from these interviews to help understand the interviewees’ general positions on interview topics, which helped inform the research, albeit superficially compared to recorded interviews. To honour the wishes of these participants and follow the lower level of details present in handwritten interview notes, we only referred to findings from these interviews as part of broad statements such as ‘X was echoed across all stakeholder groups’.

Representatives from two multilateral organizations, two hospitals, and two academics were unavailable for interview or did not respond. Had these representatives participated, perhaps our results might have been slightly less favourable for the government. However, as seen in Table 2, our final interview group was well balanced, with 9 interviewees in central government versus 15 outside of the central government–donor nexus, and 12 of the latter were from hospitals, civil society, and academia, which were generally more critical groups.

As our purposive document review was not a full systematic literature review, it is conceivable that we could have overlooked relevant studies.

Finally, the political context and EDP relationships might have evolved significantly since 2019/2020 when interviews were conducted, especially after the change of government.

## Conclusion

We identified setting aims and standards, lobbying/negotiation, providing policy/technical advice, and financing as avenues for development partner influence on domestic health financing contributions in Senegal, with a seemingly equity enhancing influence. Fungibility and intrinsic equity issues related to CBHI may, however, have limited equity gains.

We encourage stakeholders in the health financing sphere to use our framework and analysis to unpack how development partners affect domestic health financing, including equity, in other settings. This could serve as a basis for identifying dynamics that do not optimally support progress towards UHC and facilitate working towards coherent policy-making across all domains of development partner activities, which all support UHC. Our framework and analysis should be expanded and amended in other contexts as appropriate. The role of international creditors, lending, and loan conditionalities on domestic health financing in recipient countries should also be further explored, including equity implications.

## Supplementary Material

czae110_Supp

## Data Availability

The data underlying this article will be shared in anonymous form on reasonable request to the corresponding author.

## Reflexivity statement

Our author group consists of two women and three men. F.F. is from Denmark, J.B. from the UK, H.T.J. from Denmark, M.M.A. from Spain, and E.M.M. from Senegal. M.M.A. lived for >5 years in Senegal, including during the period of research. F.F. is a PhD candidate; M.M.A. is an honorary associate professor and independent research consultant, H.T.J. is an associate professor; E.M.M. is an associate professor, team lead, and a global health worker; and J.B. is a research group leader and professor. Our author group is therefore well balanced in terms of seniority, but it has a predominance of men and Western Caucasians. All data were collected and analysed by first author F.F., who as a White male of Western background is foreign to the study context in terms of culture, religion, and ethnicity and who demographically represents historical, colonial, racial, and gender-related oppression and power relations. This may have affected what interviewees may have wanted to share with him, what he may have been sensitive towards and understood, and how he may have interpreted interview data. F.F. sought to mitigate the effects of this circumstance via studying the Senegalese context extensively before conducting the research, conducting a mock interview receiving critical feedback before interviews were commenced, applying continuous reflexivity about his own effect on the research and interview participants, and asking his Senegalese interpreter for feedback after interviews.
